# Rational Approach toward COVID-19’s Main Protease Inhibitors: A Hierarchical Biochemoinformatics Analysis

**DOI:** 10.3390/ijms25126715

**Published:** 2024-06-18

**Authors:** Ruan S. Bastos, Christiane P. O. de Aguiar, Jorddy N. Cruz, Ryan S. Ramos, Njogu M. Kimani, João S. N. de Souza, Mariana H. Chaves, Humberto F. de Freitas, Samuel S. R. Pita, Cleydson B. R. dos Santos

**Affiliations:** 1 Graduate Program in Medicinal Chemistry and Molecular Modeling, Federal University of Pará, Belém 66075-110, PA, Brazil; 2 Laboratory of Modeling and Computational Chemistry, Department of Biological and Health Sciences, Federal University of Amapá, Macapa 68903-419, AP, Brazil; 3 Department of Physical Sciences, University of Embu, Embu P.O. Box 6-60100, Kenya; 4 Natural Product Chemistry and Computational Drug Discovery Laboratory, Embu P.O. Box 6-60100, Kenya; 5 Chemistry Department, Federal University of Piauí, Teresina 64049-550, PI, Brazil; 6 Laboratory of Bioinformatics and Molecular Modeling (LaBiMM), Federal University of Bahia, Av. Barão de Jeremoabo, 147, Pharmacy College, Ondina, Salvador 40170-115, BA, Brazil; hffreitas@uefs.br (H.F.d.F.); samuel.pita@ufba.br (S.S.R.P.)

**Keywords:** SARS-CoV-2, drug development, molecular docking, molecular dynamics, binding affinity

## Abstract

This study investigated the potential of selected compounds as inhibitors of SARS-CoV-2 M^pro^ through pharmacokinetic and toxicological analyses, molecular docking, and molecular dynamics simulations. In silico molecular docking simulations revealed promising ligands with favorable binding affinities for M^pro^, ranging from −6.2 to −9.5 kcal/mol. Moreover, molecular dynamics simulations demonstrated the stability of protein–ligand complexes over 200 ns, maintaining protein secondary structures. MM-PBSA analysis revealed favorable interactions between ligands and M^pro^, with negative binding energy values. Hydrogen bond formation capacity during molecular dynamics was confirmed, indicating consistent interactions with M^pro^ catalytic residues. Based on these findings, selected ligands show promise for future studies in developing COVID-19 treatments.

## 1. Introduction

Severe Acute Respiratory Syndrome Coronavirus 2 (SARS-CoV-2), the causative agent of the respiratory zoonotic disease coronavirus disease (COVID-19), was first reported in Wuhan, China, in 2019 [1]. As of 15 March 2024, the disease had claimed over seven million lives globally, with 774,834,251 confirmed cases of infection [2]. The disease is currently a global pandemic. The disease is highly contagious and mainly spread through contact with respiratory droplets from the coughing or sneezing of an infected person [2,3]. While a percentage of the cases are asymptomatic, some patients develop severe respiratory failure. The main symptoms, however, include fever, fatigue, dry cough, myalgia, and dyspnea [3].

The SARS-CoV-2 viral genome encodes several proteins. These include structural proteins such as S, N, M, and E proteins, non-structural proteins such as 3-chymotrypsin-like protease (3CL^pro^ or main protease, M^pro^), papain-like protease, helicase, and RNA-dependent RNA polymerase, as well as accessory proteins which enable the virus to evade the host immune system [4]. The main protease (M^pro^) is one of the most relied upon by the virus for replication through the hydrolysis of polyproteins at more than 11 sites to generate several functional proteins [3,4,5]. For this reason, this protease is an attractive and validated drug target. It has been proven that inhibition of this protein curtails viral replication [4,5,6].

Peptides are abundant in every living organism, and because of their unique size, they can access many targets inside the cell to which they can bind. Additionally, they possess a wide structural diversity and are not complicated to synthesize. Moreover, they show a unique mode of action with limited off-target activity and there exists advanced techniques to increase peptide half-life. There are many success stories of peptide-like inhibitors in the treatment of several diseases, such as cancer, diabetes, and autoimmune diseases [7,8,9].

Classical drug discovery methods, such as the wet lab experiments on drug–target protein interactions, are costly and time-consuming. On the other hand, in silico techniques offer a valuable alternative, allowing researchers to efficiently test their hypotheses regarding potential drug candidates, ultimately accelerating the drug development process [10]. While there are many ongoing efforts to develop vaccines that are safe and effective, until there is a solid solution, the search for effective drugs remains crucial. There is a call to fight this pandemic from all angles, employing every intervention with potential benefits.

**Figure 1:**
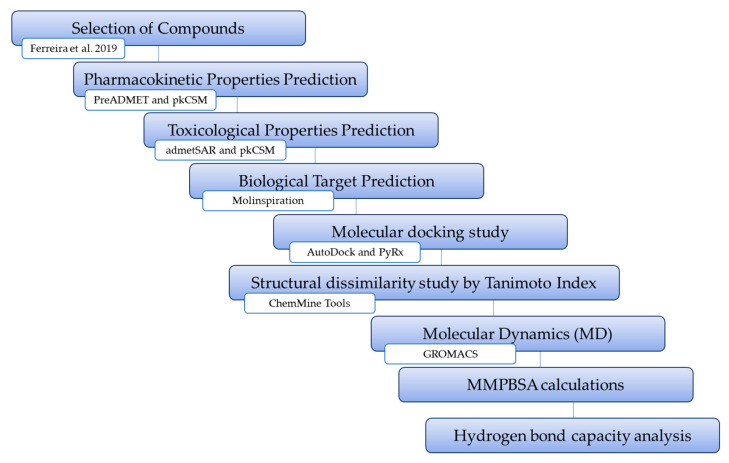
General scheme summarizing the methodological steps [7].

In our ongoing pursuit of potential antiviral compounds against SARS-CoV-2 [11,12], we employed in silico methods to identify potential COVID-19 therapeutics by targeting SARS-CoV-2 M^pro^ inhibitors. A total of 22 peptides were obtained from the literature, and their absorption, distribution, metabolism, excretion, and toxicity (ADMET) properties and drug-likeness were predicted using the Lipinski Rule of 5. Additionally, predictions were made regarding human targets for these compounds, and three compounds were selected for molecular docking studies with SARS-CoV-2 M^pro^. Subsequently, they were subjected to molecular dynamics simulation analysis. The general scheme summarizing the methodological steps in this paper is shown in Figure 1.

## 2. Results and Discussion

### 2.1. Prediction of Pharmacokinetic Properties

The in silico study of pharmacokinetic parameters is an essential tool for the discovery, selection, and development of bioactive compounds with a view to preclinical and clinical studies due to their speed, low cost, and the reduction in the number of experiments involving animals. In this work, PreADMET and pkCSM web-based tools were used for this task. PreADMET (https://preadmet.webservice.bmdrc.org/ (accessed on 20 August 2021)) is a free access web server capable of predicting ADMET properties based on the lower binding energy of molecular structures with the receptor protein. On the other hand, the pkCSM platform (http://biosig.unimelb.edu.au/pkcsm/prediction (accessed on 25 August 2021)) integrates the use of structural signatures based on graphs for the theoretical prediction of ADMET characteristics in the discovery of new drugs [13]. Table 1 shows the results of the 22 molecules and reference compounds after being subjected to the theoretical prediction.

The bioavailability of a drug administered orally is determined according to its absorption through the human gastrointestinal tract. Our analysis identified six compounds (**14**, **18**, **19**, **20**, **21**, and **22**) exhibiting exceptional HIA values, ranging from 70% to 97%. The remaining compounds displayed moderate absorption potential, with HIA values between 20% and 70%. All studied molecules, including the three reference molecules, showed median permeability for Caco2 cells ranging between 14–41 nm/s. The set of studied compounds showed skin permeability similar to the controls (FJC, lopinavir, and ritonavir). Notably, a greater permeability through the skin can be considered an unfavorable factor in drug development. Regarding distribution, **18**, **20**, **21**, and **22** are molecules that have a high affinity of binding to plasma proteins (PPB > 90%) and, therefore, are less available in their free form in circulation [13]. BBB permeation and CNS permeability are relevant factors when drugs that do not act on the brain are desired [14]. The prediction result showed poor distribution capacity across the blood–brain barrier (log BB < −1.0) and penetration to the brain (log PS < −3.0) for most compounds. In addition, all controls showed no BBB permeability and were, consequently, unable to cross into the CNS [13,14].

### 2.2. Toxicity Risk Analysis

Using computational tools to predict harmful effects on human and environmental health allows the reduction of costs and research time in the development of drugs [15]. The toxicological property end points of the compounds were calculated using the admetSAR (http://lmmd.ecust.edu.cn/admetsar2/ (accessed on 30 August 2021)) and pkCSM (http://biosig.unimelb.edu.au/pkcsm/prediction (accessed on 25 August 2021)) online tools. The results of the descriptors are summarized in Table 2.

Exposure to drugs of synthetic origin can be considered one of the risk factors for the development of cancer [15]. In this context, evaluating the adverse effects produced by these must be considered in the initial stages of the research to protect against damage to DNA or changes in cellular metabolism [15,16]. The result of this in silico study shows that none of the investigated chemical structures tested positive for carcinogenicity. The prolonged use of some medications can also induce changes in the genome with the possibility of hereditary transmission. These medications are called mutagens [17].

The Ames test assesses the mutagenic potential of drugs in strains of bacteria. Ames test calculations showed a mutagenic potential only for compounds **18**, **20**, and **22**. Drugs that do not induce injury to the liver tissue through prolonged use are desirable [18]. Based on the prediction study of the studied compounds, including the controls, only **3**, **7**, **11**, and **22** probably do not produce hepatotoxicity after chronic use. Evaluating acute oral toxicity (AOT) is necessary to determine the safest dose range of a drug candidate, guiding pre-clinical toxicological trials [18]. According to the classification criteria of Occupational Safety and Health Administration (OSHA, 2016 [19]), the value expressed as the median oral lethal dose (LD_50_) for category III varies between 50 mg/kg and 300 mg/kg.

The determination of the maximum tolerated dose (MTD) during the clinical phase study aims to ensure the safety of the drug during the treatment time for a given disease. The MTD analysis ranged from 637 to 777 mg/kg/day for most compounds. Compounds **18**, **19**, and **22**, as well as the controls, presented unsatisfactory results for this descriptor (log mg/Kg/day < 0.477). Long QT syndrome is a known consequence of blocking potassium channels encoded by the hERG gene, leading to potentially fatal ventricular arrhythmia [20]. Encouragingly, none of the tested compounds showed potential for hERG I inhibition. However, compounds **18**, **20**, **21**, and **22**, as well as all controls, have potential inhibition for hERG II.

### 2.3. Biological Target Prediction

The biological activity score of molecules has a common interpretation in the literature. If the score is >0, the molecule is active; if the score is between −5.0 to 0.0, the molecule is moderately active, and if the score is <−5.0, the molecule is considered inactive [21]. The present study was limited to evaluating only the protease-inhibiting activity of the molecules. While they are promising targets for potential drugs to combat SARS-CoV-2, it occurs because the virus has the gene replicase 229E, which encodes polyproteins that mediate all the functions required for viral replication and transcription [22]. The proteolytic processes involved in replication are mainly implemented by the proteinase M^pro^ [23] and are also related to the endosomal proteases cine B cysteine and transmembrane serine protease 2 (TMPRSS2) as possible critical components in the process of viral replication and pathogenicity [22].

The crystalline structures of these proteases have been the starting point for designing planned medicines through in silico studies, using molecular docking tools to reposition chemical entities with potential binding affinity [24]. The 22 molecules in this study showed that the target protease of structure **11** has the most likely affinity binding to proteases with a value of 46.7% and a protease inhibitor score of −0.86, indicating that the molecule is partially active for this class target (see Table 3).

Structure **10** showed an affinity percentage value of 40.0% and a biological activity score of −0.20, while structure **4** exhibited values of 33.3% and −0.04 for the class of proteases and bioactivity score, respectively. The other biological target predictions are included in the Supplementary Materials for this study (Table S1). Compounds **19** to **22** had affinity percentage values of 20.0%, 6.7%, 6.7%, and 13.3%, respectively, with activity scores of −0.06, −0.42, −0.58, and 0.16, respectively (see Table 3).

Although the human protein binding affinity values for the nine molecules involved in the study were shown to be less than the lowest value found among the controls (ritonavir = 20.0%), all compounds exhibited high or moderate inhibitory activity for the protease enzyme in the in silico analysis, thus justifying the evaluation of the inhibition of ligands against the enzyme M^pro^.

### 2.4. Molecular Docking Study

We employed the Mpro wild-type structure here (PDB ID: 6M0K) since Kaptan et al. 2022 [25] reported that all Mpro mutations, despite being distributed on the enzyme surface, were absent from its dimerization interface, highlighting that Mpro should be conserved for its viral activity.

The validation of the molecular docking was performed by comparing the crystallographic poses with obtained experimental values [25]. The Root Mean Square Deviation (RMSD) results from less than 2.0 Å to the crystallographic pose are considered acceptable [26]. The RMSD between the calculated (FJC) ligand and the crystallographic ligand displayed a value of 1.67 Å using the Discovery Studio (DS) Visualizer 17.2.0 program [27], and the visual information was confirmed by the PoseView platform [28].

**Figure 2:**
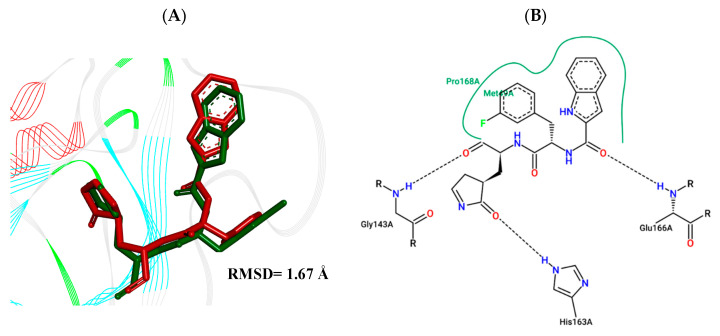
(**A**) Superposition of crystallographic ligand pose (in green) with the experimental ligand pose (in red) obtained from molecular docking validation, the blue ribbons belong to the protein structure (Mpro); (**B**) 2D interaction of the residues of molecule FJC with the target M^pro^ obtained in the validation of molecular docking.

The best pose obtained for validation is shown in Figure 2. The M^pro^ protease enzyme possesses a specific conformation around the α-helix (Glu47-Leu50) within its crystallographic structure. This conformation facilitates interaction with residue Met49. Conversely, on the β-sheet, ligands bind to the amino acids His163–Glu166 of the receptor’s active site (PDB 6M0K). The main interactions involved were of the conventional hydrogen bonding type observed in the His163 and Glu166 residues, located in the β-sheet, and Gly143 in the macromolecule loop. Within the α-helix, Met49 and Pro168 residues appear to be involved in hydrophobic and electronic interactions, respectively. These findings are consistent with previous work by Dai et al. [29], further supporting the validation of the docking protocol.

After validation, all compounds selected were coupled with binding affinity energies for the target M^pro^, varying between −6.00 to −8.40 kcal/mol. The values found for binding affinity for the study are shown in Table S2. Of the selected chemical structures, only molecule **10** showed a superior binding affinity (−8.4 kcal/mol) out of all of the control ligands used in docking (FJC: −8.2 kcal/mol; lopinavir: −6.9 kcal/mol; ritonavir: −7.2 kcal/mol). Compounds **1**, **3** to **6**, **11**, **12**, **14** to **17**, **20** and **21** exhibited binding affinities similar to or exceeding those of the control molecules, lopinavir and ritonavir. Compounds **2** and **13** displayed higher coupling energies compared to lopinavir only. The free binding energies of **7**, **8**, **9**, **18**, and **22** were low in comparison to all control ligands and only **19** had a docking score similar to lopinavir (−6.9 kcal/mol), see Table S2.

The introduction of methyl groups to the neighboring fraction of carbamoyl pyrrolidine and in the acetamide of the o-tolyl-ureido-phenyl fraction increased the binding energy compared to the pivot structure (**1**). Likewise, the spatial orientation has a considerable effect in raising the energy value, as observed in the region of connection between the carbamoyl and the pyrrolidine ring fractions. In addition, the removal of one of the groups mentioned above or their introduction can change the spatial orientation alone; however, they do not present results lower than that of compound **10**. Examples of this are demonstrated in the removal of the methyl radical from the acetamide attached to the o-tolyl-ureido-phenyl fraction (compound **6**); by the cyclization of the carbon chain in the region between the oxygen attached to the pyrrolic ring and the amide in the oxo-acid region (compound **16**); or even by changing the spatial arrangement of the carbamoyl fragment linked to pyrrole (compound **9**).

Molecular docking also allowed the identification of the main interactions involved in the coupling of the different residues linked to the active sites of the FJC (PDB ID 6M0K). Except in **18**, all ligands showed some type of interaction with the residues of the M^pro^ receptor binding site (Tables S3 and S4). After analyzing the results, an additional study was performed to investigate the structural dissimilarity of the molecules in order to select the molecules for molecular dynamics simulation (DS).

### 2.5. Structural Dissimilarity Study Using the Tanimoto Index

**Figure 3:**
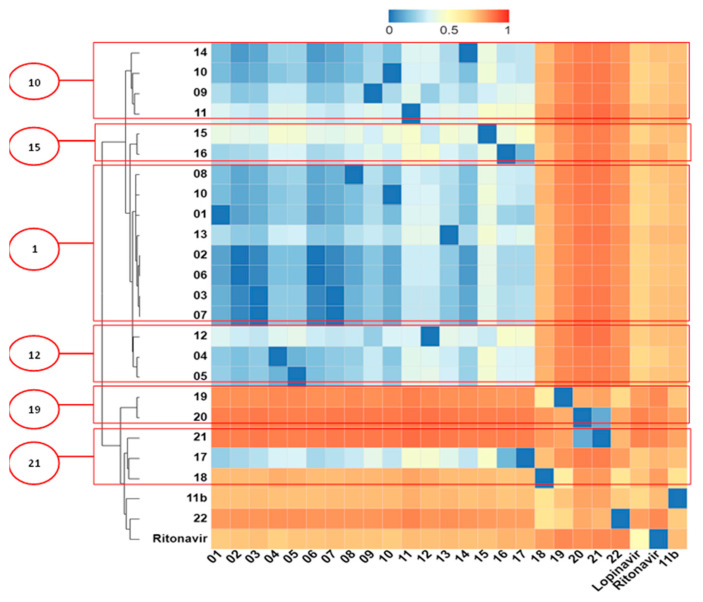
Heatmap of the hierarchical grouping between the molecules based on the Tanimoto Index (TI). This heatmap illustrates the hierarchical clustering of the ligands and the reference molecules. The clustering is based on their pairwise TI similarity. Higher TI values (darker red squares) indicate greater structural similarity between the corresponding molecules. The clustering dendrogram on the left of the heatmap depicts the hierarchical relationships between the molecules.

The heatmap of the hierarchical cluster of the molecules is illustrated in Figure 3, based on the Tanimoto Index. The similarity analysis provided valuable insights into how each fragment formed possible interactions at the binding site of the molecular targets. This analysis could also facilitate the design of molecules with increased chemical diversity. The results analysis obtained in the molecular docking and Tanimoto Index studies allowed the identification of six ligands (**1**, **10**, **12**, **15**, **19** and **21**) as potential inhibitors of M^pro^.

In Cluster 1 (molecules **9**, **10**, **11**, and **14**), a high level of structural similarity was observed with a Tanimoto Index (TI) of 0.94 with the fragment (S)-4-(((2R,3S)-1-((R)-2-carbamoylpyrrolidin-1-yl)-3-methyl-1-oxopentan-2-yl)amino)-4-oxo-3-((S)-2-(2-(4-(3-phenylureido) phenyl)acetamido)hexanamido)butanoic acid. The interactions established with the fragment are observed with the amino acid residues Thr45, Cys145, and Met165 of the hydrophobic type and Thr25 of the hydrogen bonding type. The interaction with the Met165 residue is also observed in the control ligands, according to molecular docking studies. The (S)-4-(isopropylamino)-4-oxo-3-((S)-2-(2-(4-(3-(o-tolyl) ureido) phenyl) acetamido) hexanamido) butanoic acid fragment is observed with a TI of 0.66 between ligands **15** and **16** in Cluster 2. The fragments established interactions with the residues His41, Trh45, and Glu166, all of which are classified as the hydrophobic type and Thr25 as the hydrogen bonding type.

In Cluster 3, the molecular fragment (R)-1-(((R)-2-((S)-2-(2-(4-(3-(o-tolyl) ureido)phenyl)acetamido)hexanamido)butanoyl)-D-valyl) pyrrolidine-2-carboxamide, present in the vast majority of ligands in this group, has a TI of 0.88. The interactions established by this fragment were with the residues Met165 (hydrophobic) and Gln189 (hydrogen bonding). In Cluster 4, the fragment (S)-4-(((2R,3R)-1-(((S)-sec-butyl)amino)-3-methyl-1-oxopentan-2-yl)amino)-4 -oxo-3-((S)-2-(2-(4-(3-(o-tolyl)ureido) phenyl) acetamido) pentanamido) butanoic acid was common among the ligands with a TI of 0.84. Interactions by this fragment were established between it and the amino acid residues Ans142 and Gly143 (hydrophobic) and Ser44 and Gln189 of the hydrogen bond type.

The N-methyl-2-(6-oxopyrimidin-1(6H)-yl)acetamide fragment was common among the ligands present in Cluster 5 with a TI of 0.25. The interaction related to this fragment was of the hydrogen bond type with Gly143. In Cluster 6, the fragment N-(4-methylphenyl) formamide indicated a TI of 0.14 among the ligands in the group. The region of the fragment at first does not establish any interaction directly with the target binding site, but it is a region with charged groups of free electron pairs (-NH- and =O) that help in stereochemical stabilization.

Compounds selected based on visual inspection of protein–ligand complex interactions compared to crystallographic ligands and controls (lopinavir and ritonavir), using the PoseView 2D platform [28], are shown in Table 4 and Table S4. The PoseView platform allowed the identification of residue Glu166, common to all controls. It was observed that hydrophobic interactions occur better with lopinavir and ritonavir, and that hydrogen bond interactions are most frequent on FJC and ritonavir, all located in the β-strand of M^pro^.

In addition to FJC, whose interactions were explained in the molecular docking validation, lopinavir and ritonavir were used as controls. Lopinavir interacts hydrophobically with three amino acids: two residues located in the β helix (Thr26 and Glu166) and one in the protein loop (Thr25). Still in the loop, the Asn142 binds via hydrogen bonds next to the Gly143 residue. Ritonavir, in turn, interacts through hydrophobic bonds with the amino acid Met49 in the β helix band, in the same way as Met165, but located in the β helix. The amino acids Asp187 and Leu141 interact through hydrophobic bonds in the protein loop, while the Glu166 residue, found around the β helix, interacts through both C-C and H-O bonds. Conventional hydrogen interactions are also observed in the protein loop for residues Thr25 and Gly143.

Regarding the molecules selected by the molecular anchoring study, hydrophobic interactions were observed with the Cys145 residue between the residues Asn142, Gly143, and Ser146, located in the protein loop. Hydrophobic bonds around the β-strand were located for Met165 between residues Glu166, His163, and His164 for ligand 1, similar to the bonds seen in ritonavir. In addition, hydrogen bonds with residues Ser144 and Gln189 were found in the loop region of the macromolecule.

In structure **10**, C-C-type bonds were identified in three regions of the protein: one in the β helix range for residue Met49, comprising residues Glu47, Asp48, and Leu 50, similar to ritonavir and FJC. The second for the Met165 residue, similar to ritonavir, located in the β-sheet, next to Glu166. The last, Thr45, was not found in any of the controls, located in the protein loop region next to Cys144. Hydrogen-bond-type interactions with the His163 residue were observed around the β sheet for structure **10**, similar to FJC. There was also an interaction between the hydrogen of Cys145 and the oxygen of the molecule in the loop region of the protein, between the amino acids Asn142, Ser144, and Gly146. Residues Gly143 and Thr25, located around the loop, also presented hydrogen bonds, similar to FJC and ritonavir for the first residue and ritonavir for the second, since the interaction for this amino acid in lopinavir occurs through hydrophobic interactions.

Residues Met49 and Met165 interacted through hydrophobic interactions in the α and β helices for structure **12**, respectively, with the first amino acid located between residues Glu 47–Leu50, similar to those seen in the FJC and ritonavir controls, and the binding of the Met165 residue resembled ritonavir. Although hydrophobic interactions occur in Gly143, these occur differently to the FJC and ritonavir controls, in which hydrogen bonds prevail. On the other hand, the interactions of Asn142 are similar to those found in lopinavir, while the same type of binding for residues Ser46 and Glu189, located in different parts of the macroprotein loop, are unusual for the three controls used in the study, see Table 4.

In compound **15**, hydrophobic interactions around the β helix were found between residues His41 and Met49 in the β-sheet, but only the second amino acid was similar to FJC and ritonavir in the binding form. C-C bonds were also noted in the β sheet for Glu166, being common to the lopinavir and ritonavir controls, since for FJC, the conventional hydrogen interaction prevails. In the protein loop, hydrophobic interactions occurred with Thr45. Furthermore, in the protein loop, it was possible to see H-O-type interactions with the Thr25 residue, common only to ritonavir, since for lopinavir, hydrophobic bonds were found for this amino acid.

Residues Met165, Thr25, and molecule **19** are hydrophobically linked to the β sheet and loop of the protein, in a similar way to ritonavir and lopinavir, respectively. Although there are interactions with ritonavir for Thr25, this occurs through hydrogen bonds. The amino acids Asn142 and Gly143 are linked to the loop by hydrogen bonds, with bonds similar to those seen in lopinavir for the first residue and FJC and ritonavir for the second residue.

Although ligand **21** presents interactions with residues Thr45 and Glu189, none were noticed in the controls used in the present study. The amino acid Thr45 interacted through hydrophobic bonds in the protein loop next to Cys44, while Gln189 showed hydrogen bond interactions around the loop, comprising residues Arg188 and Thr190.

After analyzing the docking results, including binding affinities and interactions in PoseView, and examining their structural similarity, we selected six ligands (**1**, **10**, **12**, **15**, **19** and **21**) to run a molecular dynamics simulation (MD). This was done to evaluate the stability of protein–ligand complexes against FJC, lopinavir, and ritonavir complexes (“positive control”).

### 2.6. Molecular Dynamics (MD) Simulations

**Figure 4:**
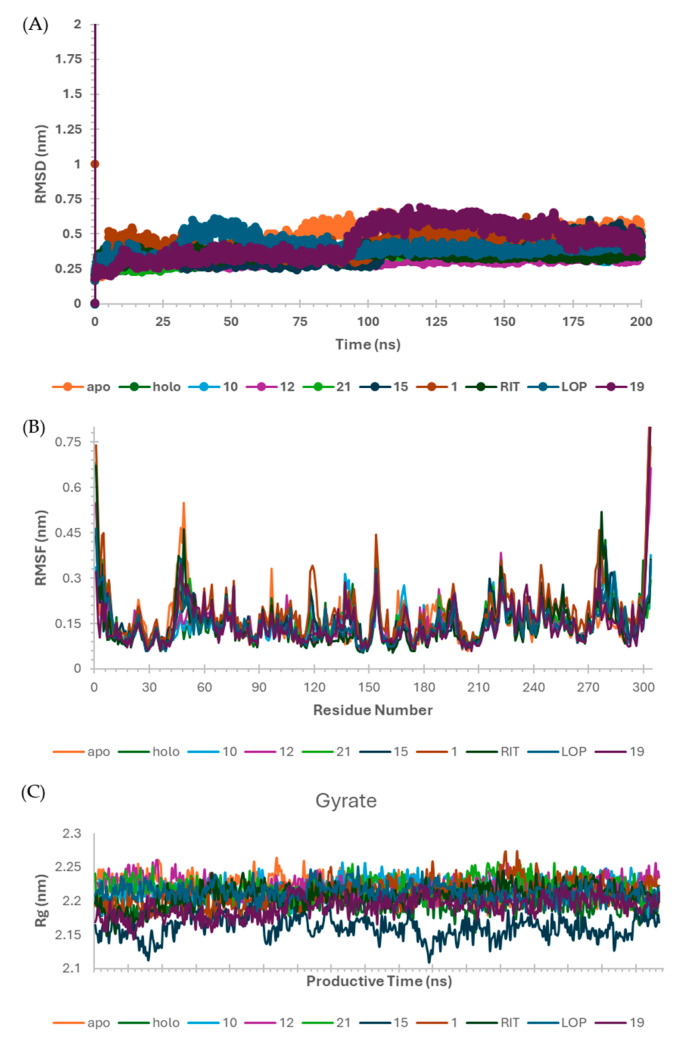
Molecular Dynamics (MD) simulation data for the main protease of SARS-CoV-2 (SARS-CoV-2 M^pro^) complexes generated for GROMACS-2021 [30,31,32,33,34,35]. (**A**) RMSD values (averaged mean ± standard deviation) for each system: apo (0.51 ± 0.03), holo (0.34 ± 0.03), 10 (0.36 ± 0.02), 12 (0.32 ± 0.02), 21 (0.34 ± 0.02), 15 (0.47 ± 0.05), 1 (0.36 ± 0.03), ritonavir RIT (0.35 ± 0.02), lopinavir LOP (0.39 ± 0.02) and 19 (0.34 ± 0.03); (**B**) RMSF and (**C**) Radius of Gyration, both from the productive phase (50–100 ns).

To understand the behavior of SARS-CoV-2 M^pro^ complexes, we analyzed them through Molecular Dynamics (MD) Simulations. These data allowed us to analyze the time influence on these interaction patterns. First, we analyzed the stability of our nine systems: apo; holo (PDB ID: 6M0K [29]—“control”); ritonavir, lopinavir (protease inhibitors, “positive control”) and the six better-ranked peptide complexes (Table 4). From our Root Mean Square Deviation (RMSD) data, we note that all systems equilibrated after 50 ns (Figure 4A). Next, we defined our productive phase into time intervals from 50–100 ns for all simulations (Figure 4B,C).

**Figure 5:**
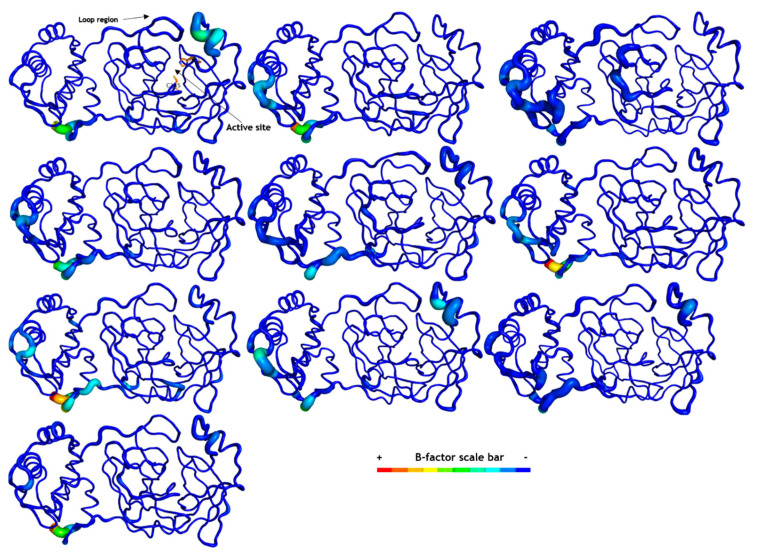
SARS-CoV-2 M^pro^ structures are shown in B-factor putty mode in PyMOL. The highest B-factor in each structure is colored in red and the lowest in dark blue, as indicated by the B-factor scale bar. The thickness of the protein backbone is also proportional to the B-factors. The catalytic residues (His41 and Cys145) are displayed as sticks only in the apo system. From the upper left corner to the right: apo, holo, **10**, **12**, **21**, **15**, **1**, ritonavir (RIT), lopinavir (LOP), and **19**. This image was generated by educational Pymol 2.4.1 [40].

Comparing the M^pro^ structure fluctuations (3D RMSF) within all complexes, we distinguished their overall protein folding stability, mainly at active sites represented by catalytic residues (His41 and Cys145, Figure 4). These data correlate with the crystallographic structure of M^pro^, where active site residues are well-solved in electron density maps with a resolution of 1.50 Å [29]. Additionally, we analyzed their secondary structure stability using the DSSP 3.1.4 module [36,37,38] installed on GROMACS-2021 [30,31,32,33,34,35]. All M^pro^ complexes maintained their secondary structure stability during our simulation time (Figure S1). Some regions presented higher fluctuations in M^pro^ structure, mostly at some loops and at the N- and C-terminals (Figure 4). The SARS-CoV-2 M^pro^ crystal had a long loop region of domain II (residues 185–200) connecting to domain III, and this region is highly variable as pointed out by the superposition of **12** M^pro^ crystal structures [29]. Our MD data revealed that these loops on domain II fluctuated more than other secondary protein structures, corroborating with this experimental study. Previous results also cited the interface region among M^pro^ protomers composed of domain II residues as unstable [23,29,39] and these data were also visualized in our simulations (Figure 5).

Many studies described M^pro^ subsites expanding from S6 to S1 with a catalytic site located on S1 [39,41,42,43]. Based on these results, we analyzed the interaction pattern on the SARS-CoV-2 M^pro^ of NatProDB derivatives compared with a crystallographic ligand (FJC) and two protein inhibitors (lopinavir—LOP and ritonavir—RIT). Additionally, we evaluated both the hydrogen bond pattern, through the GROMACS hbond module [44] and the HbMap2Grace program [45], and the molecular Surface Area using the SurfinMD program [44].

The Molecular Dynamics (MD) data for hydrogen bond (H-bond) analysis showed that peptide compounds made more interactions than the crystallographic ligand (FJC) and two positive controls (lopinavir and ritonavir) in S1, S4, and S1′ M^pro^ subsites, especially on another new site that was recently discovered [12] (Cys44, Ser46, Glu47, and Asn119) (Table S5). Since these interaction patterns could favor their inhibitory behavior, we analyzed them in detail.

Initially, we defined and calculated the hydrogen bond capability for our ligands, Hbond_capac._, during MD simulation. This measure could aid in quantifying how “tightly” the ligands interact with M^pro^ since Hbond_capac_. ≥ 1 means that each ligand’s hydrogen bond atoms (donors and acceptors) interacted with protein residues.

**Figure 6:**
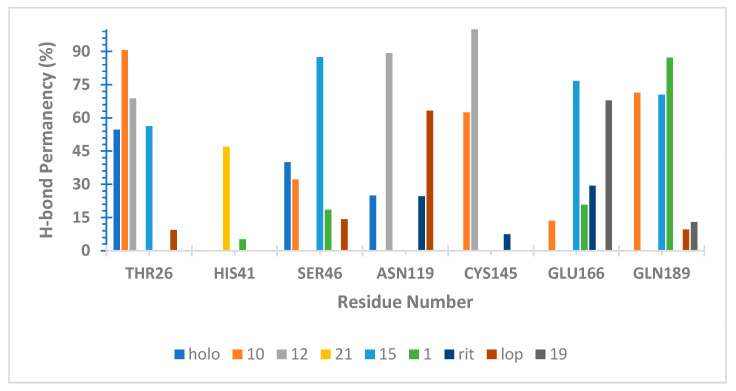
Hydrogen bond stability on SARS-CoV-2 Mpro complexes calculated by HbMap2Grace [45] during the productive phase of Molecular Dynamics simulation (50–100 ns).

Our results from the MD productive phase correlate well with the estimated binding energy obtained before docking results (Table 4), i.e., the ligands with the best docking energies presented higher Hbond_capac._, which reveals to us that a hydrogen bond is a favorable interaction for developing M^pro^ inhibitors. Additionally, we noted that our ligand accessed distinct sub-sites on SARS-CoV-2 M^pro^, interacting beyond the catalytic sites (S1) (Figure 6).

The H-bond pattern presented interactions with His41 and Cys145 (catalytic residues); Thr25 and 26 from S1′; Gly143 and Ser144 from the canonical oxyanion role on S1 [41]; Glu166 from S1; and Gln189 and Thr190 from S4 [40,42,43,46]. Since an H-bond is considered as the “driving force” for M^pro^ inhibition, we could map and analyze them through our MD simulations.

**Figure 7:**
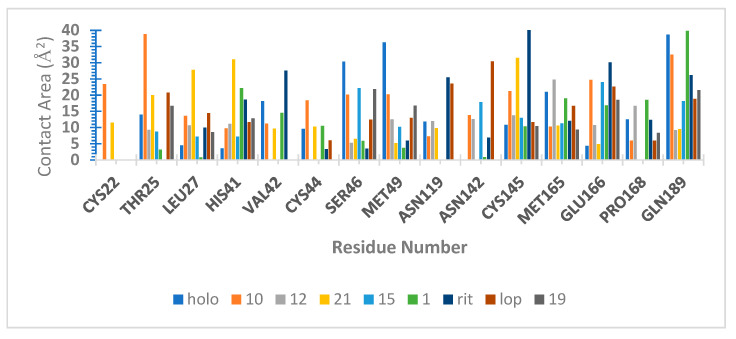
Surface Molecular Area (Å^2^) of SARS-CoV-2 Mpro complexes calculated by SurfinMD [44] during the productive phase of the Molecular Dynamics simulation (50–100 ns).

We also calculated the atomic contacts involving SARS-CoV-2 M^pro^ and peptide compounds (Figure 7). The contact surface area revealed additional interactions with apolar residues on the same sites described before [12,23,40,42,43,46]: S1′ (Thr25, 26, Leu 27, Cys145), S2 (His41, Thr45, Ser46, Asp48, Met49), S4 (Met165, Glu166, Leu 167, Gln189), and S5 (Pro168).

Additionally, we calculated the binding free energy of all M^pro^ complexes through MM-PBSA methods. The binding energy (ΔE_binding_) calculated using the solvent-accessible surface area shows that all compounds interacted favorably with M^pro^. Since these values are directly correlated to interacting protein residues, we decided to assess which amino acids presented better contact with ligands. Decomposition energy analyses of these residues enabled the selection of residues near the ligand (<5 Å) during the MD simulation and those that participate actively in complex stabilization (ΔE_binding_ > ± 5 kJ/mol), as shown in Figure S2.

We noted that ligand interaction with catalytic residues (His41 and Cys145) are highly favorable (negative values), as expected for reversible inhibitors. This behavior remains for two residues of the new binding site (Arg188 and Gln189), first described in this work. Other residues (Ser46, Glu47, Asp48, and Gln189) do not interact favorably with ligands (positive values—Figure S2). This could be related to their position on the M^pro^ site, i.e., their side chains are pointed outwards instead of towards the active site cavity [23,40,46].

## 3. Materials and Methods

### 3.1. Selection of Compounds

The search for new inhibitors based on drug repositioning has become a strategy of great interest in the pharmaceutical industry in combating COVID-19 [11,46]. The 22 compounds (see Supplementary Table S6) involved in this study were obtained from the PubChem server (https://pubchem.ncbi.nlm.nih.gov/ (accessed on 10 August 2021)). These compounds were from the study by Ferreira et al. [7], which was based on the best inhibitory activity values obtained from studies by Dattoli et al. [47] and Liu et al. [48], where peptidomimetic inhibition assays on Jurkat cell adhesion to immobilized CS-1 and ligand binding affinity were studied by inhibiting α4β1-mediated cell adhesion. IC_50_ values indicate the concentration required to inhibit a biological process by half, thus providing a measure of the potency of the antagonist drug [49].

**Figure 8:**
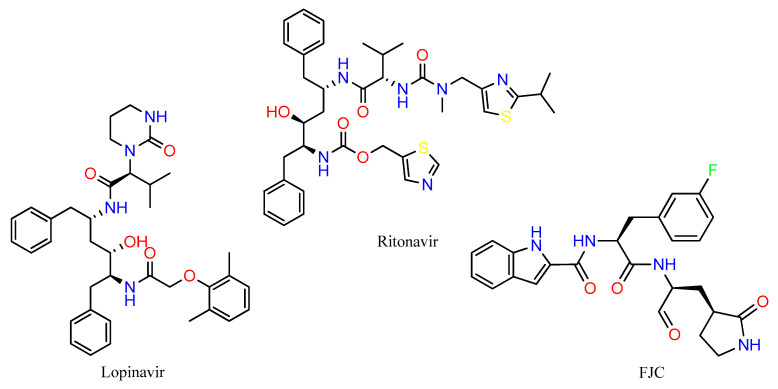
The 2D chemical structures of the control compounds used in this study.

M^pro^ protease inhibitors lopinavir and ritonavir administered together for the treatment of COVID-19 [50,51] were used as controls and accessed from PubChem. Both drugs are part of the therapeutic scheme used to treat Human Immunodeficiency Virus (HIV) and they have reduced the viral load of β-coronavirus and improved the clinical condition in patients with the disease [50]. The high specificity of lopinavir through its active protease site contrasts with its reduced oral bioavailability and extensive hepatic metabolism. In this sense, ritonavir is co-administered to increase the plasma concentration of lopinavir and improve its antiviral activity. The crystallographic structure of the FJC (Figure 8—PDB ID: 6M0K) compound was used as a reference due to its strong inhibitory activity against SARS-CoV-2 (IC_50_ = 0.040 ± 0.002 µM) [29].

After selection, it was necessary to optimize the structures. To minimize errors in interaction models arising from the use of non-bioactive conformations in conformationally flexible molecules, all chemical structures were converted to the *.mol format in the OpenBabel 2.3.2 tool [52]. Subsequently, the structures were optimized in HyperChem 6.03 software [53]. The method selected was Molecular Mechanics with the MM+ force field [54].

### 3.2. Pharmacokinetic Properties Prediction

The online servers PreADMET (https://preadmet.webservice.bmdrc.org/ (accessed on 20 August 2021)) and pkCSM (https://biosig.lab.uq.edu.au/pkcsm/ (accessed on 25 August 2021)) were used to predict the absorption, distribution, metabolism, and excretion (ADME) properties of each compound [55]. The platforms calculated different pharmacokinetic properties, such as Human Intestinal Absorption (HIA), Caco2 cell permeability in vitro (PCaco2), skin permeability (PSkin), Plasma Protein Binding (PPB), permeation of the CBrain/CBlood barrier (Blood–Brain Barrier—BBB) and Central Nervous System permeability (CNS) [56]. The molecular structures saved in the *.mol format were used to obtain toxicological information using the PreADMET server, while the ChemSketch program, version 11.02 [57], was used to generate SMILE codes for each compound, which were then submitted to the pkCSM server.

### 3.3. Toxicological Properties Prediction

The toxicity of the compounds was predicted on two online servers: admetSAR (http://lmmd.ecust.edu.cn/admetsar2/ (accessed on 30 August 2021)) and pkCSM (https://biosig.lab.uq.edu.au/pkcsm/ (accessed on 25 August 2021)). These two servers make predictions based on the structural similarities found in comparison to the compounds in their respective databases [58,59]. The two online platforms obtained information related to carcinogenicity, mutagenicity, hepatotoxicity, acute oral toxicity (AOT), Maximum Tolerated Dose human (MTD), and hERG I and II inhibition.

### 3.4. Biological Target Prediction

The SMILES codes obtained through ChemSketch 11.02 [57] were subsequently submitted to Swiss Target Prediction (https://www.swisstargetprediction.ch/ (accessed on 7 September 2021)) and Molinspiration (https://www.molinspiration.com/ (accessed on 10 September 2021)) for assessment of target prediction and protease inhibition activity, respectively. As a criterion, molecular docking studies were carried out only with compounds that showed protease binding affinity equal to or greater than the values of the controls employed in this study and the probability of protease inhibition with active or partially active activity.

### 3.5. Molecular Docking Study

Molecular docking simulations were performed using AutoDock 4.2/Vina 1.1.2 software [60] and the PyRx interface version 0.8 [61]. In this study, the Lamarckian Genetic Algorithm (LGA) was used, with standard parameters of the genetic algorithm (with a population size of 150), a maximum number of evaluations of 250,000, a maximum number of generations of 27,000, and a crossing rate of 0.8.

The molecular docking study was initially validated to verify whether the coupling parameters specified in the input file for the docking method are reasonable and capable of recovering the structure and interactions of the known complex [59]. For this purpose, the crystallographic ligand was removed from the receptor–ligand complex and subsequently replaced with the original receptor with the coupling parameters validated by calculating the Root Mean Square Deviation (RMSD) to obtain the best spatial conformation, following the previously validated protocol adopted by our research group [62,63,64].

The structure of the target protein (PDB ID: 6M0K) was downloaded in PDB format from the RCSB Protein Data Bank (https://www.rcsb.org/ (accessed on 12 September 2021)) and used in the preparation of the ligand and receptor through the Discovery Studio (DS) Visualizer 17.2.0 program [27]. After validation, the compounds selected for the molecular docking study were coupled. Their binding affinity energies were calculated for the SARS-CoV-2 protease receptor (6M0K) [29]. The DS Visualizer 17.2.0 program was used for the calculation of RMSD and PoseView [65] was utilized to generate the interactions between the inhibitors and the receptor with standard parameters.

### 3.6. Structural Dissimilarity Study Using the Tanimoto Index

Hierarchical clustering methods were used to select the molecules with ChemMine Tools (https://chemminetools.ucr.edu/ (accessed on 22 September 2021)), in which the structural similarity measures of the clusters were calculated from atomic descriptors between each molecular pair, which generated a similarity matrix based on unique and common characteristics observed between molecules using the Tanimoto Index (0 = less similar to 1 = greater similarity) [66]. In the subsequent grouping steps, the similarity matrix was converted into a distance matrix by subtracting the similarity values from 1. The search for similarity by ChemMine Web Tools allowed the structural comparison of ligands and their grouping according to similarity based on the Tanimoto Index [66,67,68].

### 3.7. Molecular Dynamics (MD) Simulation on SARS-CoV-2 Mpro

GROMACS-2021 software [30,31,32,33,34,35], available from the National Center for High-Performance Computing in São Paulo (CENAPAD-SP), was used for Molecular Dynamics (MD) simulations. The following parameters were used: time = 200 ns, 1 atm, 310 K, pH 7.5, and GROMOS54A7 force-field updated [26]; PME for electrostatic treatment [64] with 1.0 nm of cut-off for non-covalent interactions; periodic boundary conditions (PBC) with 1 ps writing steps and the SPC/E water model [69] in a dodecahedral simulation box. Na^+^ and Cl^−^ ions were added to maintain the physiological salt concentration (0.15 M) and to neutralize the residual system charge at pH = 7.5, as described previously [70,71]. At first, the system was energy-minimized (steepest descent/conjugate gradient) until forces reached ≤10 kJ·mol^−1^ nm^−1^.

Then, a pre-equilibrium simulation step (heavy atoms’ position restrained for 1 ns) was performed under T = 310 K and system pressure maintenance at 1 atm (NPT ensemble) with a V-rescale thermostat [72] and a Berendsen barostat [73]. To better simulate biological conditions (pH = 5.5) [74], all pka residues were determined by PROPKA 3.1 [75]; all acidic and basic residues were charged, and all histidine residues were neutral. An unrestrained simulation was performed for 200 ns for all systems with the SETTLE algorithm [76] for solvent bonds and LINCS [77] for other bonds. The topology coordinates of the catechol ligands (peltatoside and maritimein) were built in the Automated Topology Builder (ATB) version 3.0 server (http://compbio.biosci.uq.edu.au/atb/, accessed on 9 May 2022) [78].

### 3.8. MMPBSA Calculations

In addition to molecular docking and molecular dynamics simulation, molecular mechanics/Poisson–Boltzmann surface area (MM-PBSA) was applied to determine the thermodynamical stability of the SARS-CoV-2 M^pro^ complexes and to investigate the contribution of each residue in the binding pocket. The MM-PBSA were calculated using the g_mmpbsa tool [59]. This method calculates the binding energy (∆E_binding_), which represents the average of two energetic terms: potential energy in the vacuum (ΔE_MM_) and the free solvation energy (∆G_solvation_), as described by Equation (1).

∆E_binding_ = ∆E_MM_ + ∆G_solvation_, (1)

The molecular mechanic (MM) energy term (ΔE_MM_) is calculated based on electrostatic (ΔE_elec_) and van der Waals (ΔE_vdW_) interaction components according to molecular mechanics force-field parameters [59]. The solvation energy is computed based on polar solvation energy (ΔG_pol_), using the Poisson–Boltzmann (PB) equation [62], and non-polar solvation energy (ΔG_nonpol_), estimated from the solvent-accessible surface area (SASA), including repulsive and attractive forces between solute and solvent that are generated by cavity formation and van der Waals interactions [59]. To decompose the binding energy, ΔE_MM_, ΔG_pol_, and ΔG_nonpol_ were first separately calculated for each residue and then summed to obtain the contribution of each residue to the binding energy [59].

The energy components E_MM_, G_pol_, and G_nonpol_ of the M^pro^ (apo) and M^pro^–peptide complexes were calculated for 500 snapshots extracted every 0.1 ns from the production trajectories between 50 and 100 ns. E_MM_ was calculated using the LJ and Coulomb potentials. To calculate G_pol_, a box was generated using the extreme coordinates of the molecular complex in each dimension. The box was then expanded in each dimension by 1.5-fold to obtain a coarse-grid box (cfac = 1.5). A finer grid box was then placed within the coarse grid box, extending 5 Å (fadd = 5) from the complex’s extreme coordinates in each direction. The ionic strength of 0.150 M NaCl with radii of 0.95 and 1.81 Å for sodium and chloride ions, respectively, was used for all G_pol_ calculations. The values for the vacuum (vdie), solvent (sdie), and solute (pdie) dielectric constants were taken as 1, 80, and 2, respectively. The solvent radius was set to 1.4 Å, and the temperature was set to 310 K. The linear PB equation was solved using the APBS 3.4.1 program [62]. G_nonpolar_ was calculated using solvent-accessible surface area (SASA) nonpolar models using a surface tension of (gamma) 0.0226778 KJ/(mol A^2^) and a probe radius of 1.4 Å.

### 3.9. Hydrogen Bond Capacity Analysis

We also calculated the hydrogen bond capacity (Hbond_capac_.) as previously defined [34]:

(2) Hbondcapac.=Hbond∑HBD,HBA

where <Hbond> is the average hydrogen bond number during the MD simulation, ∑HBD, HBA is the sum of the hydrogen bond donor (HBD) and acceptor (HBA) of the molecule.

## 4. Conclusions

In this comprehensive study of the pharmacokinetic, toxicological, and molecular interactions of the compounds studied, along with molecular docking and molecular dynamics simulations, we conclude that the selected ligands show potential as inhibitors of SARS-CoV-2 M^pro^. The results of docking simulations revealed favorable binding affinity between the ligands and the active site of M^pro^, with binding energy values ranging from −6.2 to −9.5 kcal/mol. Furthermore, molecular dynamics simulations indicated the stability of the protein–ligand complexes over 200 ns of simulation, with maintenance of the protein’s secondary structures.

MM-PBSA analysis revealed negative binding energy values for all ligands, indicating favorable interactions between the ligands and M^pro^. Hydrogen bonding capacity during molecular dynamics simulation was also analyzed, demonstrating consistent interactions of the ligands with the catalytic residues of M^pro^, such as His41 and Cys145. Based on these results, the selected ligands show potential for future drug development studies against COVID-19.

## Figures and Tables

**Table 1:** Prediction of pharmacokinetic properties of investigated structures and controls.

Structure	HIA [a]	PCaco2 [a]	PSkin [a]	PPB [a]	BBB [b]	CNS [b]
1	60.287	20.933	−2.638	77.409	−2.277	−3.520
2	63.552	20.925	−2.566	81.291	−2.343	−3.619
3	66.583	20.925	−2.509	83.779	−2.231	−3.592
4	63.540	20.913	−2.579	79.224	−2.267	−3.533
5	67.464	20.912	−2.471	78.844	−2.295	−3.669
6	63.552	20.925	−2.566	81.291	−2.343	−3.619
7	66.583	20.925	−2.509	83.779	−2.231	−3.592
8	40.555	20.837	−2.585	68.971	−2.338	−3.898
9	66.583	20.796	−2.528	81.117	−2.264	−3.625
10	66.573	20.933	−2.510	83.616	−2.388	−3.529
11	51.168	14.396	−2.413	78.180	−1.521	−3.677
12	54.588	20.776	−2.527	83.263	−2.262	−3.643
13	69.365	20.964	−2.473	84.498	−2.297	−3.521
14	79.926	20.032	−2.501	82.428	−2.517	−3.593
15	60.302	19.484	−2.659	69.612	−2.224	−3.751
16	57.071	20.988	−2.938	66.840	−2.257	−3.921
17	56.760	21.004	−2.606	66.391	−2.293	−3.854
18	94.928	23.600	−2.942	100.000	−0.053	−2.051
19	96.829	22.372	−3.657	89.676	0.211	−1.943
20	97.181	23.469	−4.150	95.434	−1.262	−2.336
21	96.937	21.930	−4.167	96.221	−1.265	−2.446
22	94.143	41.601	−2.137	92.115	−0.205	−2.529
FJC	85.571	19.359	−4.323	90.905	−0.992	−3.347
Lopinavir	93.802	24.605	−2.520	89.712	−0.830	−2.935
Ritonavir	93.192	35.883	−2.617	86.385	−1.665	−3.295

[a] PreADMET (https://preadmet.qsarhub.com/ (accessed on 20 August 2021))—HIA: Human Intestinal Absorption (%); PCaco2: Caco2 cell permeability in vitro (nm/s); PSkin: Skin Permeability (cm/h); PPB: Plasma Protein Binding (%); [b] pkCSM (http://biosig.unimelb.edu.au/pkcsm/prediction (accessed on 25 August 2021))—BBB: Blood–Brain Barrier permeation (log BB); CNS: Central Nervous System permeability (log PS).

**Table 2:** Toxicological prediction of selected structures and controls.

Structure	Carcino [a]	Ames [a]	Hepato [a]	MTD [b]	hERG I [b]	hERG II [b]
1	-	-	Yes	0.731	No	No
2	-	-	Yes	0.695	No	No
3	-	-	No	0.672	No	No
4	-	-	Yes	0.645	No	No
5	-	-	Yes	0.737	No	No
6	-	-	Yes	0.695	No	No
7	-	-	No	0.672	No	No
8	-	-	Yes	0.741	No	No
9	-	-	Yes	0.659	No	No
10	-	-	Yes	0.697	No	No
11	-	-	No	0.637	No	No
12	-	-	Yes	0.665	No	No
13	-	-	Yes	0.775	No	No
14	-	-	Yes	0.698	No	No
15	-	-	Yes	0.660	No	No
16	-	-	Yes	0.666	No	No
17	-	-	Yes	0.656	No	No
18	-	+	Yes	−0.614	No	Yes
19	-	-	Yes	−0.091	No	No
20	-	+	Yes	0.795	No	Yes
21	-	-	Yes	0.777	No	Yes
22	-	+	No	0.320	No	Yes
FJC	-	-	Yes	0.426	No	Yes
Lopinavir	-	-	Yes	−0.297	No	Yes
Ritonavir	-	-	Yes	0.096	No	Yes

[a] admetsar (http://lmmd.ecust.edu.cn/admetsar2/ (accessed on 30 August 2021))—Carcino: carcinogenesis; Negative (−); Positive (+); Ames: Ames test; Hepato: Hepatotoxicity; [b] pkCSM (http://biosig.unimelb.edu.au/pkcsm/prediction (accessed on 25 August 2021))—MTD = Maximum Tolerated Dose human (log mg/Kg/dia); hERG I: hERG I inhibitor; hERG II: hERG II inhibitor.

**Table 3:** Results of the biological target prediction of selected structures and controls.

Structure	Affinity Binding to Proteases [a] (%)	Protease Inhibitor [b]
1	20.0	0.04
2	13.3	−0.06
3	20.0	−0.27
4	33.3	−0.04
5	6.7	−0.07
6	13.3	−0.06
7	20.0	−0.27
8	26.7	−0.13
9	20.0	−0.20
10	40.0	−0.20
11	46.7	−0.86
12	20.0	−0.30
13	13.3	−0.34
14	20.0	−0.10
15	26.7	−0.02
16	20.0	0.24
17	18.0	0.14
18	8.0	0.18
19	20.0	−0.06
20	6.7	−0.42
21	6.7	−0.58
22	13.3	0.16
FJC	26.7	0.70
Lopinavir	26.7	0.42
Ritonavir	20.0	0.35

[a] Swiss Target Prediction (https://www.swisstargetprediction.ch (accessed on 7 September 2021)); [b] Molinspiration cheminformatics Prediction of Bioactivity (https://www.molinspiration.com/ (accessed on 10 September 2021)).

**Table 4:** Result of binding energy (kcal/mol) and 2D interaction of the selected compounds and controls with the surrounding residues obtained in the molecular docking study.

Ligand	Binding Energy (kcal/mol)	2D Interactions of Residues
1	−7.70	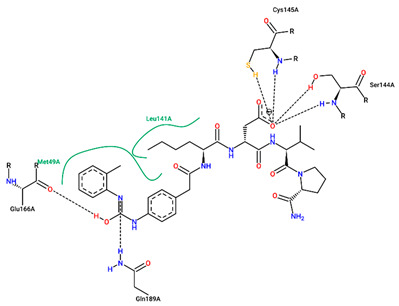
10	−8.40	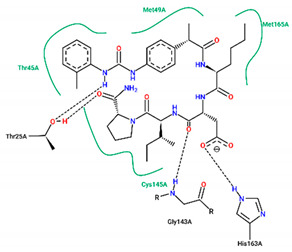
12	−7.90	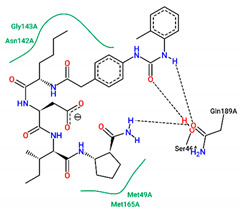
15	−7.70	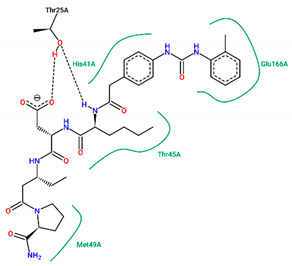
19	−6.90	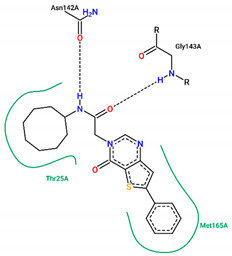
21	−7.80	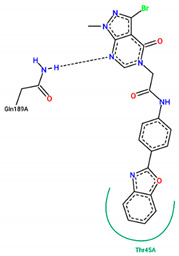
FJC	−8.20	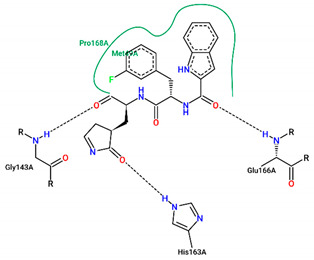
Lopinavir	−6.90	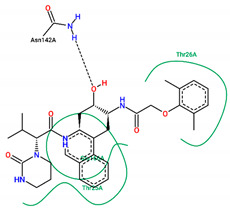
Ritonavir	−7.20	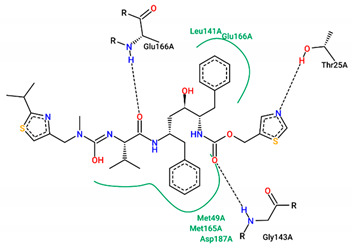

## Data Availability

Data is contained within the article and Supplementary Materials.

## Supplementary Materials

The supporting information can be downloaded at: https://www.mdpi.com/article/10.3390/ijms25126715/s1.
