# Supplementary material for: Rational Approach toward COVID-19’s Main Protease Inhibitors: A Hierarchical Biochemoinformatics Analysis
Source: Int J Mol Sci. 2024 Jun 18;25(12):6715. doi: 10.3390/ijms25126715 (PMC11204165; doi:10.3390/ijms25126715)
Supplement: Supplementary file 1 [file ijms-25-06715-s001.zip › ijms-2988241-supplementary.pdf]

Table S1. Target prediction results for investigated compounds 1-22[a].

**Target**	**Compounds**																					
**Protease**	**1**	**2**	**3**	**4**	**5**	**6**	**7**	**8**	**9**	**10**	**11**	**12**	**13**	**14**	**15**	**16**	**17**	**18**	**19**	**20**	**21**	**22**
Caspase-1		√	√	√	√	√	√	√	√	√	√	√			√	√	√	√				
Caspase-2								√			√								√			√
Caspase-3		√	√	√	√	√	√	√	√	√	√	√			√	√	√	√				
Caspase-4		√	√	√		√	√	√	√	√	√	√	√	√	√	√	√	√				
Caspase-5		√	√	√		√	√	√	√	√	√	√	√	√	√	√	√	√				
Caspase-6				√							√	√										
Caspase-7		√	√			√	√	√	√	√		√			√	√	√	√				
Caspase-8		√	√	√		√	√	√	√	√		√			√	√	√	√				
Caspase-9		√	√	√		√	√		√	√	√	√			√			√				
Caspase-10		√	√	√		√	√	√	√	√	√	√	√	√	√	√	√	√				
Chymase			√				√				√									√	√	
Chymotrypsin C			√	√			√		√			√			√	√				√	√	
Cathepsin (B and K)	√																					
Cathepsin D	√							√														
Cathepsin G										√												
Cathepsin L	√																		√			√
Cathepsin K																			√		√	√
Cathepsin S	√																		√		√	√
Complement factor B								√														
Complement factor D																					√	
Thrombin		√	√	√		√	√		√	√			√	√	√	√	√	√			√	
Granzyme B		√		√		√				√						√	√					
Endothelin-converting enzyme 1					√						√											
Coagulation factor VII											√	√										
Coagulation factor VII/tissue factor		√		√	√	√		√		√		√			√	√	√	√				
Carboxypeptidase B					√			√	√						√	√						

Carboxypeptidase B2 isoform A					√						√											
Plasma kallikrein											√											
Plasminogen									√			√			√							
Coagulation factor XI					√						√											
Coagulation factor IX					√															√		
Matrix metalloproteinase 3		√	√	√	√	√	√	√	√	√		√	√	√	√	√	√	√			√	
Matrix metalloproteinase 2		√	√	√	√	√	√	√	√	√		√	√	√	√	√	√	√				
Matrix metalloproteinase 1		√	√			√	√		√			√	√	√	√			√			√	
Matrix metalloproteinase 14																				√	√	
Matrix metalloproteinase 13											√								√		√	
Matrix metalloproteinase 12											√									√		
Matrix metalloproteinase 11		√				√																
Matrix metalloproteinase 10																					√	
Matrix metalloproteinase 9																					√	
Matrix metalloproteinase 8																						
Calpain 1	√										√											
Epoxide hydratase	√										√		√	√					√		√	√
Epoxide hydrolase 1	√																					
ADAMTS5				√				√			√									√		
ADAMTS4				√	√			√			√											

Lysosomal protective protein															√	√	√	√			
Leucine aminopeptidase								√													
Beta-chymotrypsin		√	√	√		√	√			√							√				
Beta-secretase 1	√	√	√			√	√	√	√	√		√			√	√	√	√			√
Beta-secretase 2	√	√	√			√	√	√	√			√			√		√	√	√		
Gamma-secretase	√																				√
Pepsin A		√		√	√	√				√		√	√	√				√			
Renin		√	√			√	√	√	√	√		√			√		√	√			
Carboxypeptidase N, catalytic subunit											√										
Thrombin and coagulation factor X	√														√						
Neprilysin (by homology)	√																				
Dipeptidyl peptidase I													√	√							
Dipeptidyl peptidase IV													√	√							
Leukotriene A4 hydrolase																			√		
Leukocyte elastase													√	√							√
Prolyl endopeptidase													√	√							
Furin													√	√							
Angiotensin-converting enzyme													√	√							
ADAM9													√	√							
Subtilisin/kexin type 5													√	√							
Subtilisin/kexin type 6													√	√							
Prohormone convertase 1													√	√							

Trypsin I		√	√	
Proteasome Macropain subunit MB1				√
Cathepsin (V and K)				√
Tissue-type plasminogen activator				
Bone morphogenetic protein 1	√			

[a] Swiss Target Prediction to protease inhibition activity.

Table S2. Binders selected based on binding affinity.

Structure	Binding Affinity to proteases[a] (%)
**1**	-7.70
**2**	-7.00
**3**	-7.80
**4**	-7.20
**5**	-7.90
**6**	-8.00
**7**	-6.70
**8**	-6.80
**9**	-6.30
**10**	-8.40
**11**	-7.60
**12**	-7.90
**13**	-7.10
**14**	-7.20
**15**	-7.70
**16**	-8.00
**17**	-7.60
**18**	-6.80
**19**	-6.90
**20**	-7.80
**21**	-7.80
**22**	-6.20
**FJC**	-8.20
**Lopinavir**	-6.90
**Ritonavir**	-7.20

Table S3. Residue interactions with selected binders and controls in the PoseView Tool.

Ligands	Thr2 4	Thr2 5	Thr2 6	His 41	Thr4 5	Ser4 6	Met 49	Phe1 40	Leu1 41	Asn1 42	Gly1 43	Ser1 44	Cys1 45	His1 63	Met1 65	Glu1 66	Pro1 68	Asp1 87	Gln1 89	Thr1 90	Gln1 92
1																					
2																					
3																					
4																					
5																					
6																					
7																					
8																					
9																					
10																					
11																					
12																					
13																					
14																					
15																					
16																					
17																					
18*																					

19
20
21
22
FJC
Lopinavir
Ritonavir

* No interactions found by the PoseView interaction model.

Green: common residue with all controls; Blue: common residue with 2 controls; Orange: common residue with 1 control; Red: residue not found in any of the controls.

Table S4. 2D interactions of compounds and controls with the surrounding residues.

Structure	Binding Energy (kcal/mol)	2D interactions of residues
**1**	-7.70	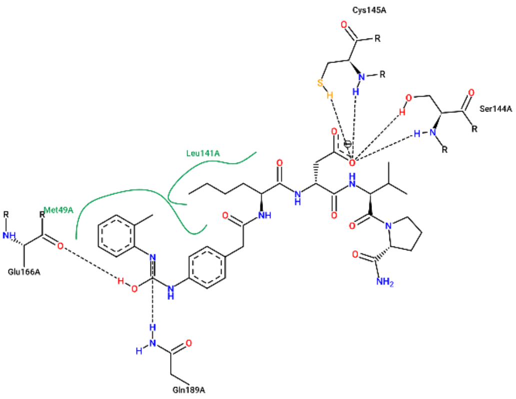
**2**	-7.00	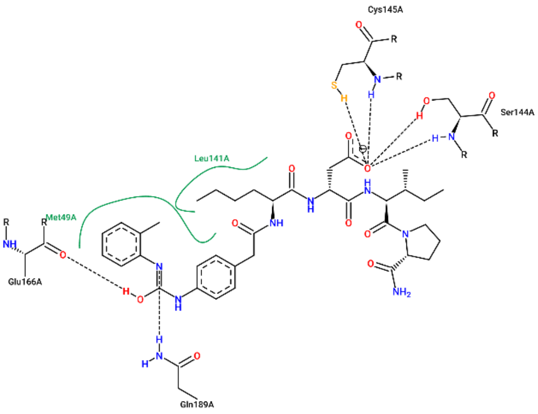
**3**	-7.80	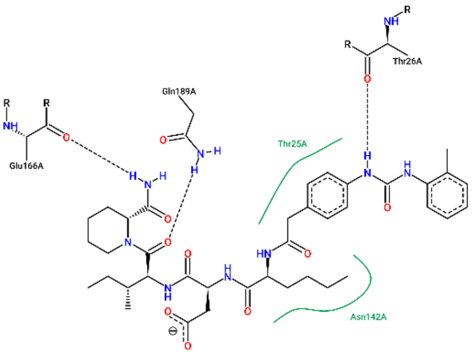
**4**	-7.20	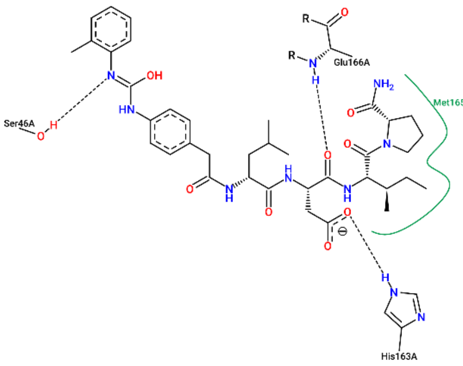

**5**	-7.90	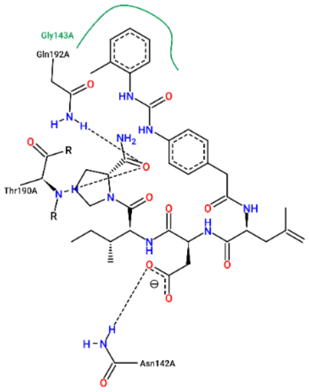
**6**	-8.00	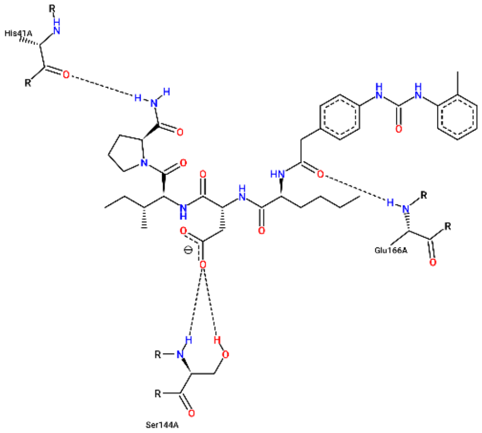
**7**	-6.70	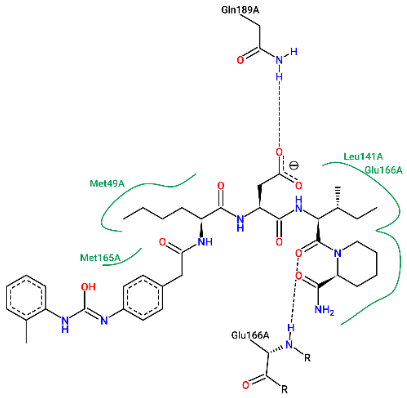
**8**	-6.80	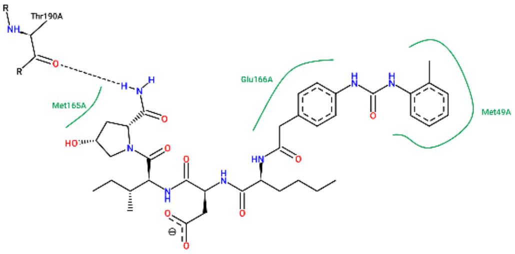

<table>
<tr><td>9</td><td>-6.30</td><td>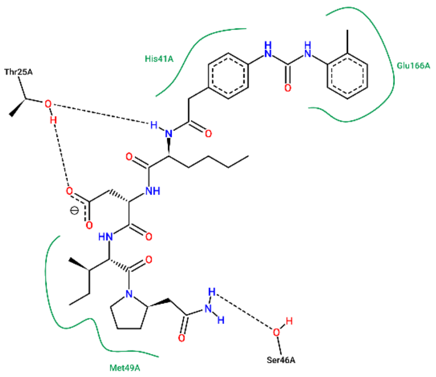
</td></tr>
<tr><td>10</td><td>-8.40</td><td>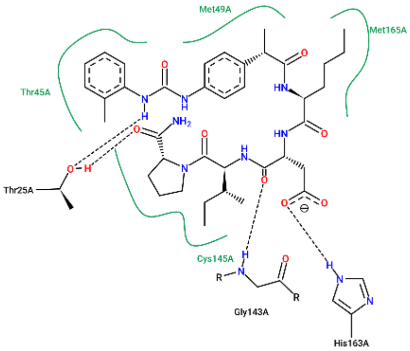
</td></tr>
<tr><td>11</td><td>-7.60</td><td>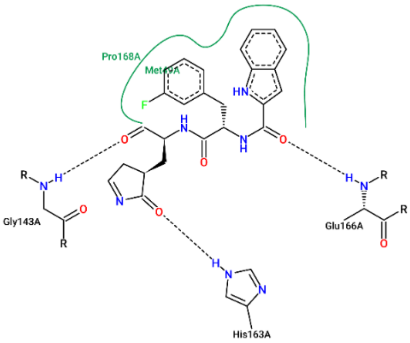
</td></tr>
<tr><td>12</td><td>-7.90</td><td>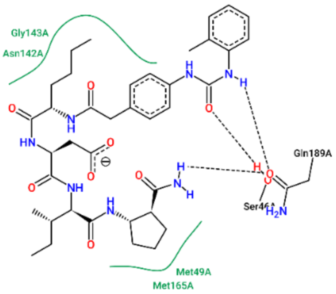
</td></tr>
</table>

**13**	-7.10	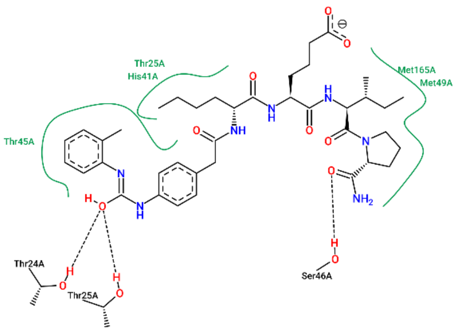
**14**	-7.20	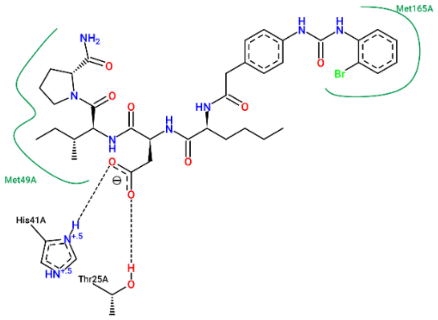
**15**	-7.70	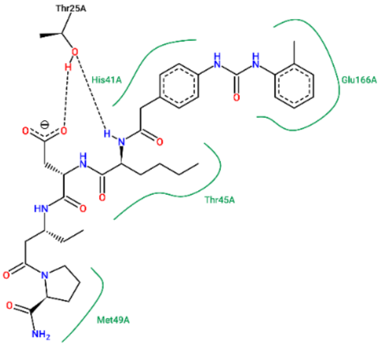
**16**	-8.00	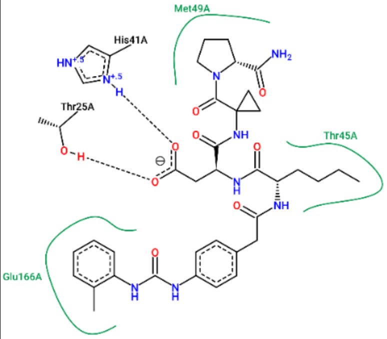

**17**	-7.60	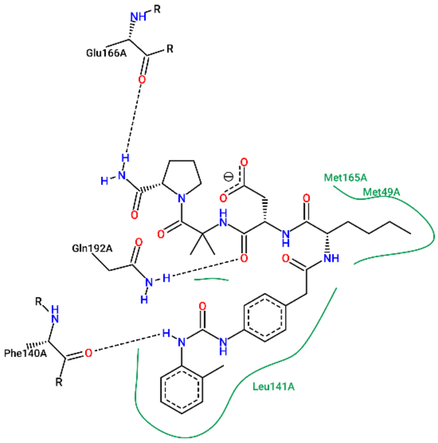
**18**	-6.80	No interactions found by the PoseView interaction model
**19**	-6.90	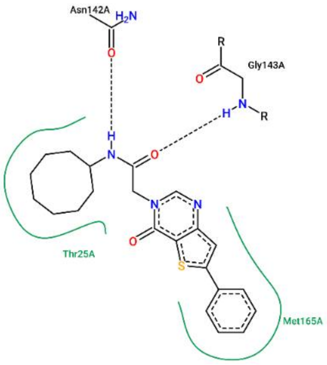
**20**	-7.80	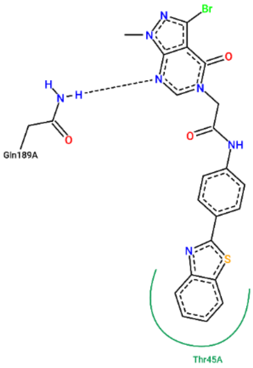
**21**	-7.80	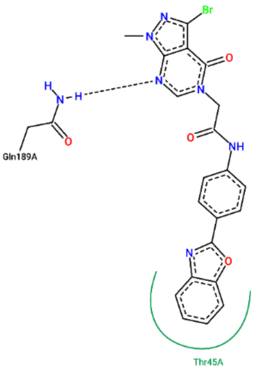

**22**	-6.20	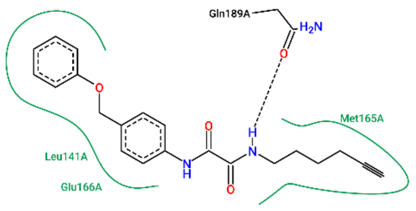
**FJC**	-8.20	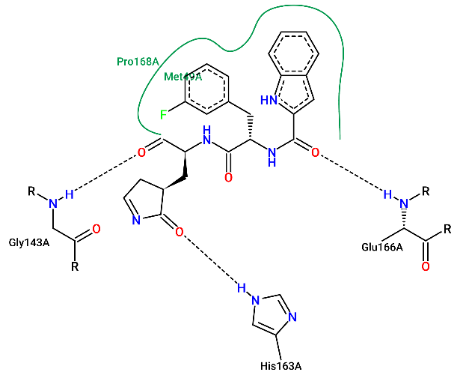
**Lopinavir**	-6.90	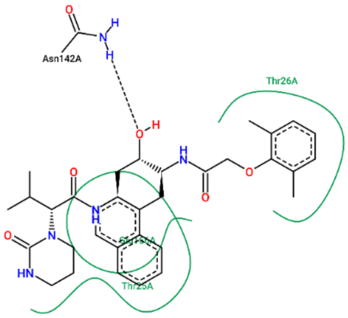
**Ritonavir**	-7.20	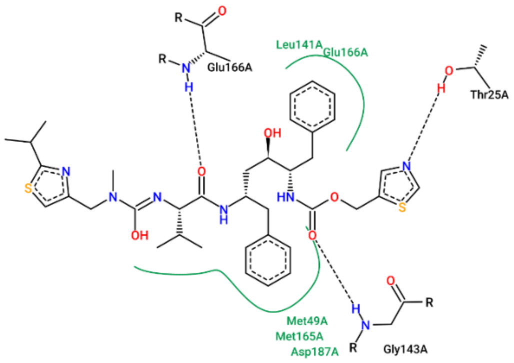

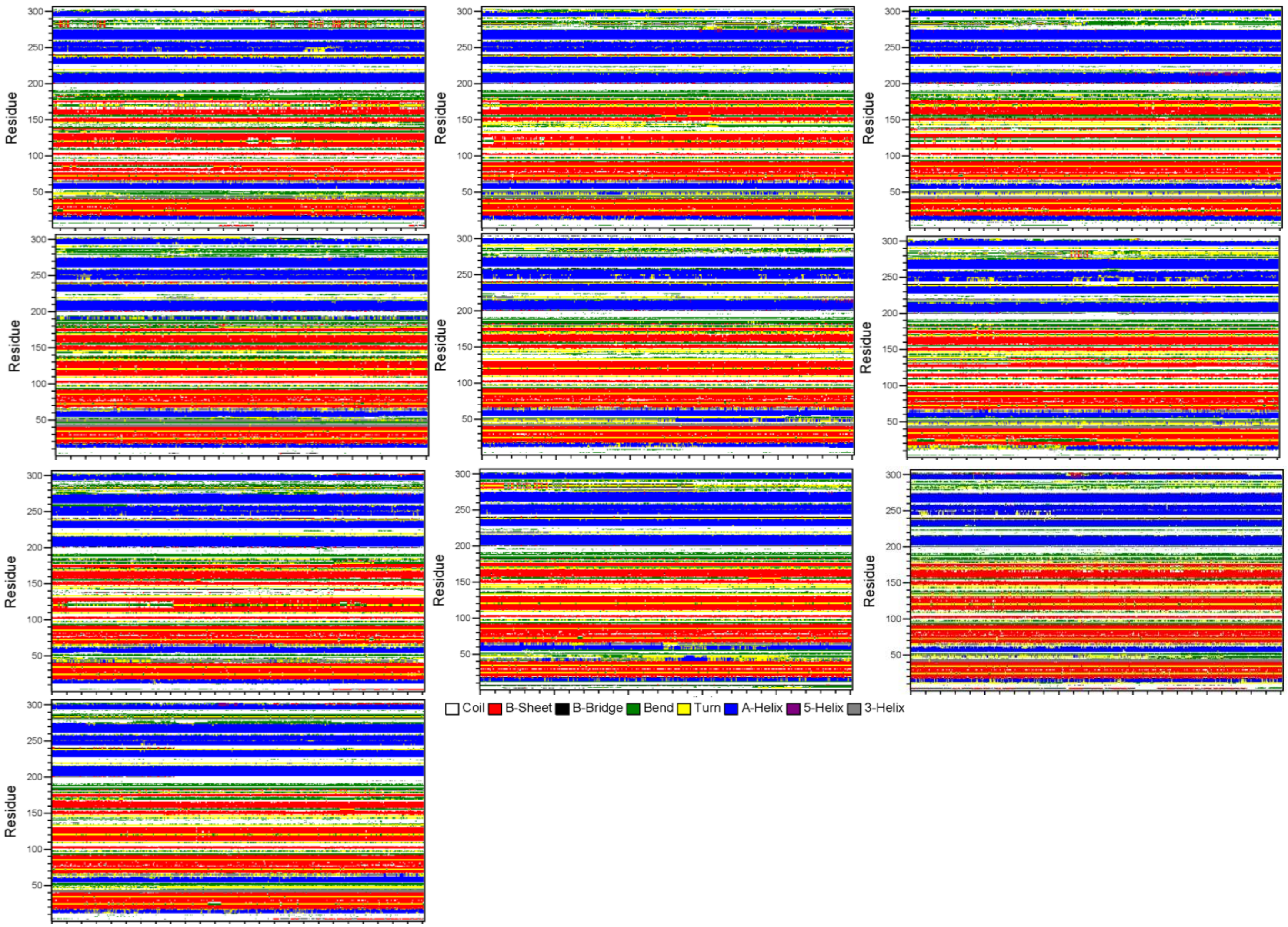

Figure S1. Productive phase (50-100ns) Secondary structure evaluation of SARS-CoV-2 Main protease (SARS-CoV-2 Mpro) complexes by DSSP 3.1.4 module installed on GROMACS-2021. From upper left corner to the right: apo, holo, 10, 12, 21, 15, 1, ritonavir (RIT), lopinavir (LOP) and 19.

Table S5. Molecular Surface Area and Hydrogen bond interactions with SARS-CoV-2 Main protease (Mpro) active site residues complexed with holo (PDB ID: 6M0K, “conrtol”); ritonavir, lopinavir (“positive controls”) and best docked peptide ligands.

**Mpro subsites**	**holo**		**10**	**12**	**21**	**15**	**1**	**RIT**	**LOP**	**19**
	**Residue**	**Contact Area (A2)**								
S1	HIS41	3.6	9.8	11.1	31.0	7.2	22.2	18.6	11.7	12.8
	ASN142	0.0	13.8	12.6	31.5	17.8	0.8	6.9	30.4	0.0
	CYS145	10.9	21.2	13.7	10.6	13.0	10.4	40.1	11.7	10.4
S2	VAL42	18.2	11.2	0.0	9.6	0.0	14.5	27.5	0.0	0.0
S3	MET49	36.3	20.2	12.5	5.2	10.2	3.7	6.0	13.0	16.8
S4	MET165	21.0	10.3	24.7	10.6	11.3	19.0	12.1	16.6	9.3
	GLU166	4.3	24.7	10.7	4.9	24.0	16.8	30.1	22.6	18.5
	PRO168	12.5	6.0	16.6	0.0	0.0	18.5	12.4	6.0	8.4
	GLN189	38.7	32.5	9.2	9.5	18.1	39.8	26.2	18.8	21.5
S1'	CYS22	0.0	23.4	0.0	11.5	0.0	0.0	0.0	0.0	0.0
	THR25	14.0	38.8	9.3	20.0	8.7	3.2	0.0	20.8	16.7
	LEU27	4.5	13.6	10.6	27.8	7.2	0.8	9.9	14.4	8.6
New	CYS44	9.6	18.4	0.0	10.3	0.0	10.5	3.3	6.0	0.0
	SER46	30.3	20.2	5.3	6.5	22.2	5.8	3.5	12.4	21.8
	ASN119	11.8	7.2	12.0	9.8	0.0	0.0	25.5	23.5	0.0
SAS SURFinMD (Å^2)		187.0	323.8	261.2	171.9	214.3	252.5	254.1	228.4	146.7

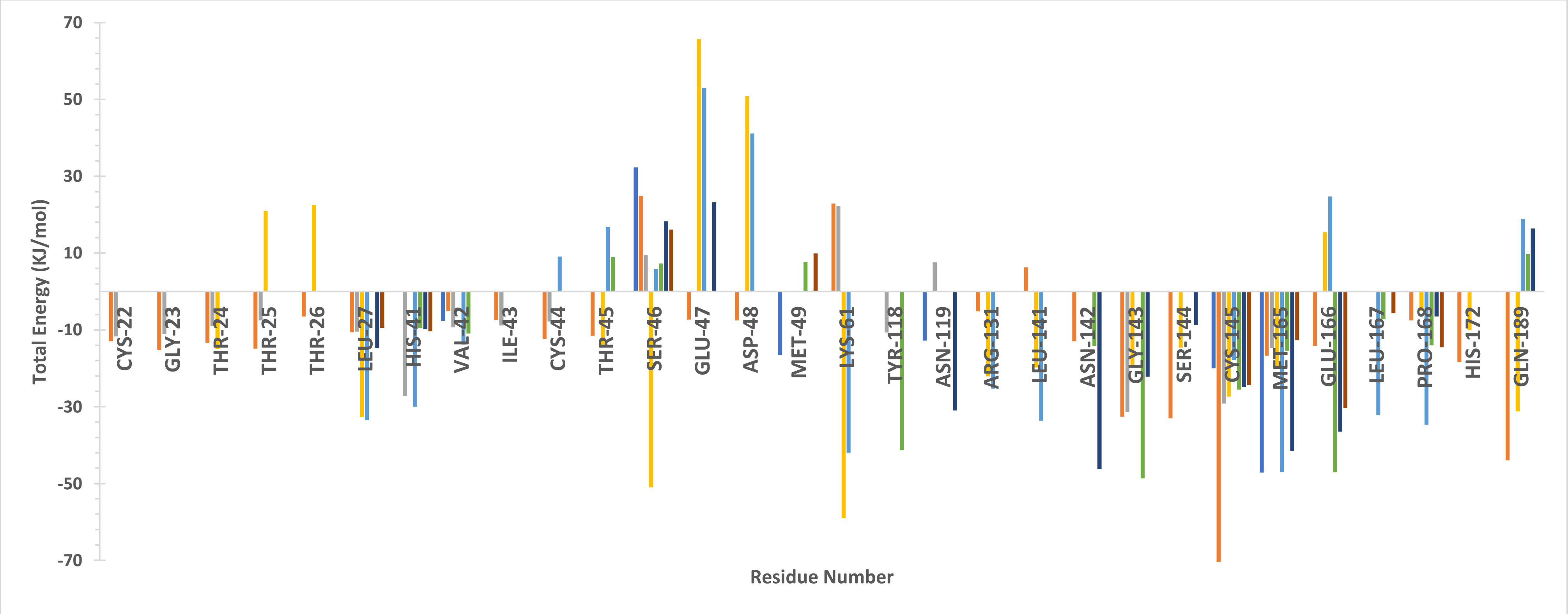

Figure S2. Residue contributions to the binding energy of SARS-CoV-2 Mpro complexes. The main residues with energy interaction (ΔEbinding > ± 5 kJ/mol) were highlighted. For Mpro sub-site definitions see the text.

International Journal of *Molecular Sciences*

MDPI

Table S6. 2D Structures of the selected compounds.

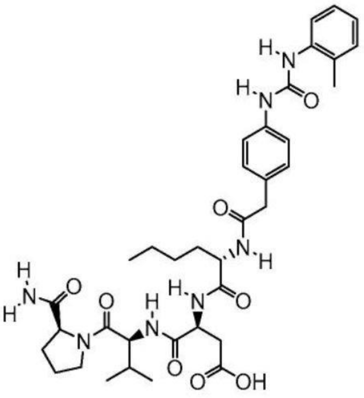 **1**	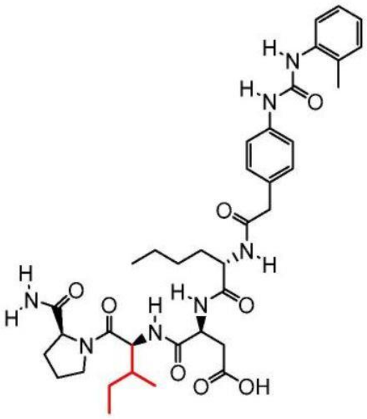 **2**
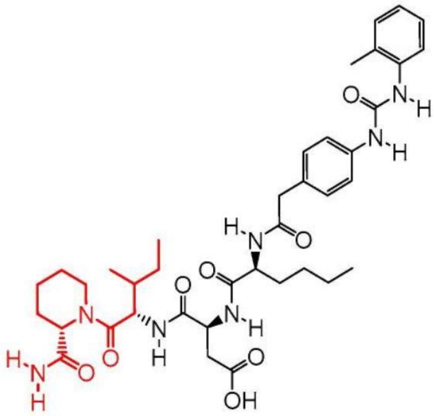 **3**	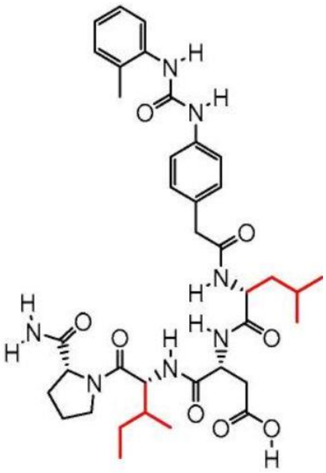 **4**
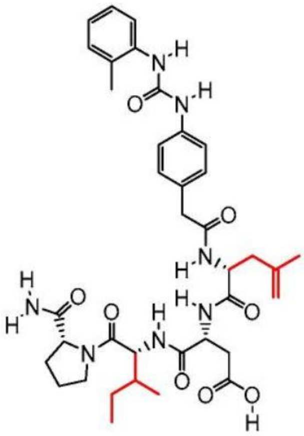 **5**	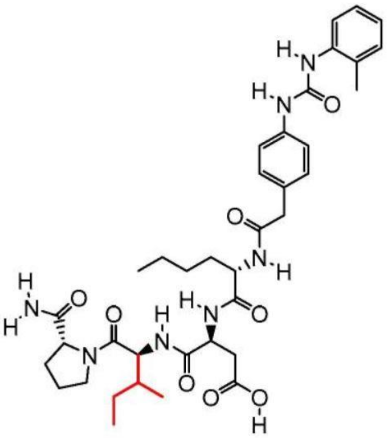 **6**

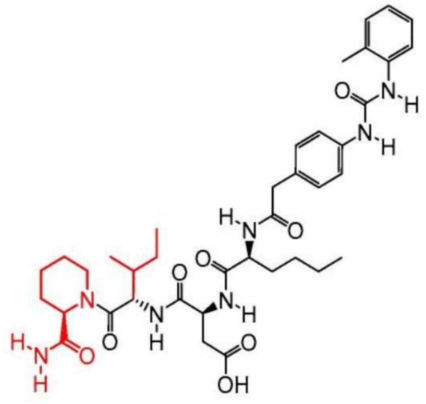<br>**7**	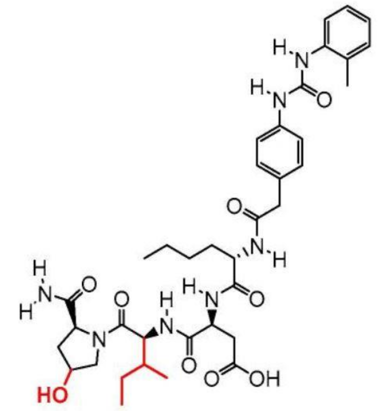<br>**8**
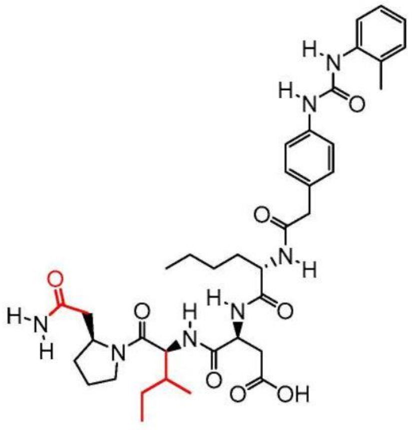<br>**9**	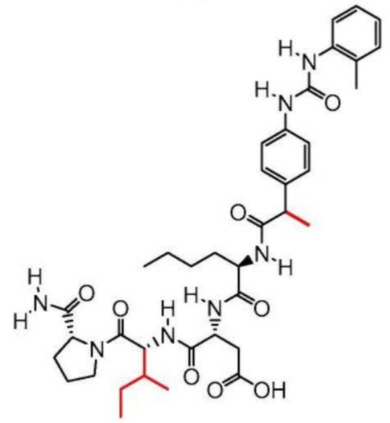<br>**10**
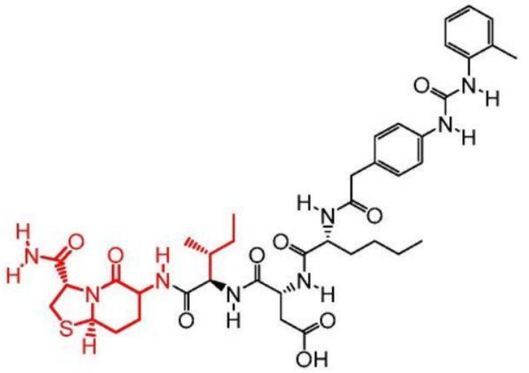<br>**11**	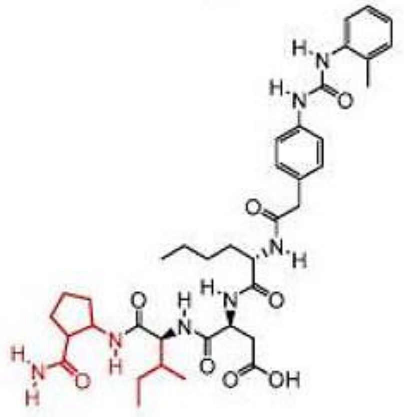<br>**12**

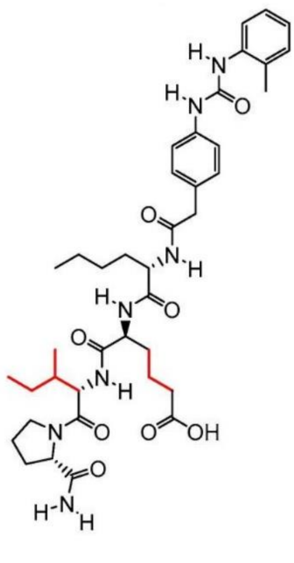 13	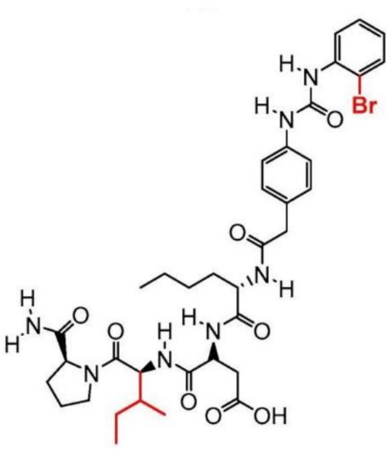 14
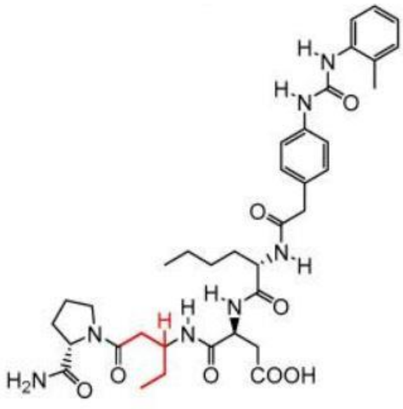 15	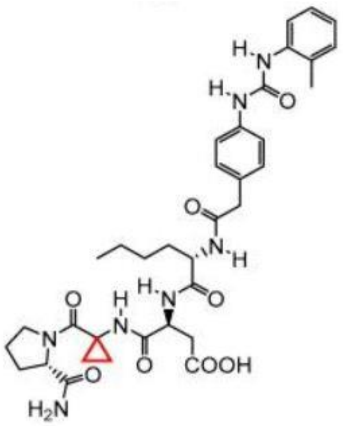 16
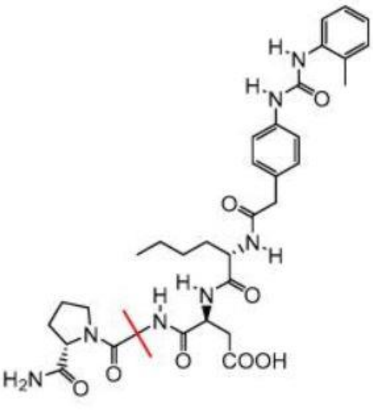 17	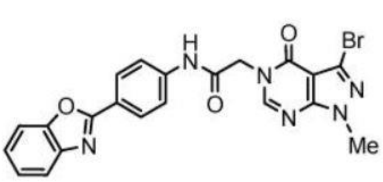 18(21)
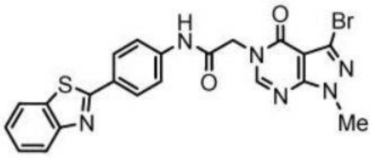	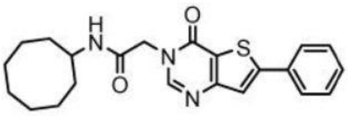

19	20(19)
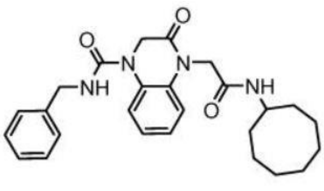<br>21	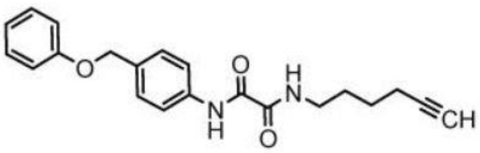<br>22
